# Lifetime history of head injury is associated with reduced perivascular space number in acute mild traumatic brain injury

**DOI:** 10.1093/braincomms/fcae314

**Published:** 2024-09-17

**Authors:** Kiersten J Garcia, Grace Brolly, Daniel Ng, Maria Bederson, Pedro Martinez, Mark D Whiting

**Affiliations:** Stephens Family Clinical Research Institute, Carle Health, Urbana, IL 61801, USA; Carle Illinois Advanced Imaging Center, Carle Health, Urbana, IL 61801, USA; Carle Illinois College of Medicine, Urbana, IL 61801, USA; Carle Illinois College of Medicine, Urbana, IL 61801, USA; Carle Illinois College of Medicine, Urbana, IL 61801, USA; Department of Neuroscience, Wartburg College, Waverly, IA 50677, USA; Stephens Family Clinical Research Institute, Carle Health, Urbana, IL 61801, USA; Carle Illinois Advanced Imaging Center, Carle Health, Urbana, IL 61801, USA; Carle Illinois College of Medicine, Urbana, IL 61801, USA

**Keywords:** traumatic brain injury, perivascular space, glymphatic system, MRI

## Abstract

Traumatic brain injury impairs function of the glymphatic system, a perivascular network involved in waste clearance. Enlarged perivascular spaces visible on MRI are an emerging biomarker of glymphatic function. This study characterized enlarged perivascular spaces in acute head injury with 7 T MRI. Healthy controls (*n* = 8) and patients (*n* = 11) with mild traumatic brain injury underwent MRI within 7 days of injury and were evaluated for lifetime history of head injury, neurobehavioral symptoms and sleep disturbances. MRI-visible perivascular spaces were quantified and assessed according to published criteria. The number of enlarged perivascular spaces was significantly higher in traumatic brain injury patients than controls (*P* = 0.015). Among healthy controls, 6/8 scored ‘none’ or ‘mild’ on the perivascular space rating scale, while 10/11 patients scored ‘moderate’, ‘frequent’ or ‘severe’. There was an inverse relationship between perivascular space number and number of lifetime head injuries. Patients with more prior head injuries exhibited fewer enlarged perivascular spaces (*P* = 0.014). These results indicate that mild head injury results in acute alterations in perivascular space number, and this effect is mediated by previous head injury history. Enlarged perivascular spaces may reflect a glymphatic response that is diminished after multiple head injuries, although this will require further study.

## Teaser

Garcia *et al*. report that patients with mild traumatic brain injury show evidence of acute alterations in perivascular space number compared to healthy controls, as characterized with 7 T MRI within 1 week of injury. Lifetime exposure to head injury was inversely related to the number of enlarged perivascular spaces.

## Graphical Abstract

**Graphical Abstract:**
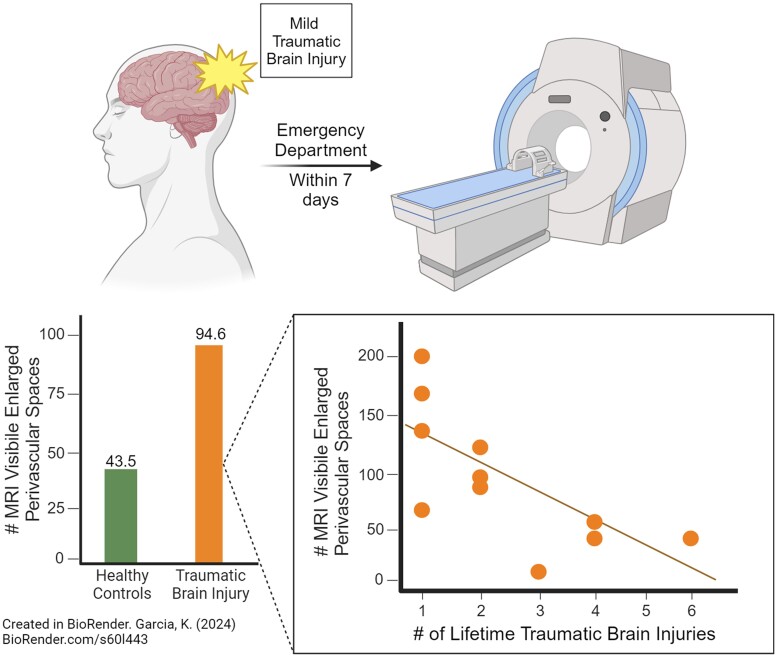

## Introduction

The glymphatic system is a network of perivascular spaces (PVS) that facilitates clearance of interstitial solutes and metabolic waste products through CSF-interstitial fluid (ISF) exchange.^1^ Glymphatic clearance is impaired after acute neurological insult, as has been shown in animal models of traumatic brain injury (TBI) and stroke.^2-4^ In humans, a number of studies have used MRI-visible enlarged perivascular spaces (ePVS) as surrogate markers of glymphatic function. Typically, ePVS are most evident in deep white matter structures such as the centrum semiovale. PVS are thought to represent fundamental elements of the glymphatic system which may become enlarged under pathological conditions. CSF-ISF exchange is highly dependent on the astroglial aquaporin-4 (AQP4) water channel, which is densely localized to perivascular astrocytic endfeet.^3^ Following TBI, there is a loss of perivascular localization of AQP4,^5^ and genetic deletion of AQP4 impairs glymphatic clearance and promotes tau pathology.^3^ Further pointing to the role of AQP4 in ePVS is a recent human study showing that CSF AQP4 levels independently predict the number of ePVS.^6^ Although the diagnostic and prognostic significance of ePVS remains to be seen, several studies have examined ePVS burden in conditions where glymphatic function is thought to be impaired, including Alzheimer’s disease,^7^ multiple sclerosis,^8^ cerebral small vessel disease^9^ and aging.^10^

ePVS have long been known to be a characteristic feature of mild TBI (mTBI).^11^ More recently, studies have begun to elucidate the nature of ePVS in mTBI and the relationship to neurobehavioral and clinical outcome, as well as how ePVS burden may interact with injury factors such as repetitive head injury and TBI-induced sleep impairment.^12-14^ At the same time, research in other populations has shown that ePVS may be an extremely sensitive and dynamic neuroimaging biomarker, influenced by individual factors such as sex, age, hypertension, cholesterol levels, white matter hyperintensities and genetics and even environmental factors including time of day.^15^ However, it is currently unclear what causes ePVS to develop and how ePVS dynamics change over time in response to mTBI. Therefore, studies that hope to elucidate the role of ePVS in the pathophysiology of mTBI should control for as many of these factors and health conditions as possible. The aim of the current study is to examine ePVS during the acute stage of mTBI in a young, otherwise healthy cohort of patients with mTBI and healthy controls, excluding possible alternative explanations for the presence of ePVS. We further sought to characterize the relationship between lifetime history of mTBI and development of ePVS during the acute stage of injury.

## Materials and methods

### Participants

All study procedures described here were approved by the Institutional Review Board at Carle Foundation Hospital. Patients presenting to the emergency department at a level I trauma center between November 2021 and June 2023, with either documented trauma to the head or suspicion of head injury triggering a clinical CT, were screened for possible enrolment. Patient medical records were screened under a partial HIPPA waiver. Patients were included in this study if they were male or female age 18–65, met the VA/DoD criteria for mTBI,^16^ were able to receive 7 T MRI within 7 days of injury and were able to provide written, informed consent in English or have a legally authorized representative provide consent. Patients were excluded if they had evidence of moderate or severe TBI, penetrating brain injury, were pregnant, unable to complete study assessments or had any contraindication to awake 7 T MRI without contrast. We further excluded patients who had a medical history of conditions associated with a high ePVS, including migraine or chronic daily headache, any neurological disorder, diagnosed sleep disorders such as obstructive sleep apnoea or insomnia and hypertension. Healthy control participants were recruited via word of mouth and flyers from the local community. In addition to the exclusion criteria applied to mTBI patients, healthy controls were excluded for lifetime history of TBI of any severity.

### Clinical and neurobehavioral data collection

Clinical and demographic variables were collected from the patient medical record, or by self-report from healthy control participants. CT images and reports were available for all mTBI patients and were collected from the medical record. All participants completed the Neurobehavioral Symptom Inventory and the Pittsburgh Sleep Quality Index. The OSU-TBI short form was used to determine lifetime exposure to TBI in patients and healthy controls.

### MRI acquisition and image analysis

All scans were performed on a Siemens Magnetom Terra 7 T (Siemens Healthcare, Erlangen, Germany) with an 8-transmit channel/32-receive channel head coil in parallel transmit mode. Dielectric pads (Multiwave Imaging, Marseille, France) were used to improve signal loss commonly seen in the temporal lobe and cerebellum at ultra-high field strength. Sequences included localizer, MP2RAGE, T2 FLAIR, SWI and T2* GRE. All sequences were obtained without contrast agent. T2* GRE imaging parameters relevant to the current analysis were as follows: echo time, 17.8 ms; repetition time, 3290 ms; slice thickness/number of slices, 1.5/85; flip angle, 25 degrees; acquisition matrix, 420 × 420; in-plane resolution, 0.5 × 0.5; acceleration factor, GRAPPA 2; and acquisition time, 10 min. The total duration of the research scan was ∼50 min. MRI-visible ePVS, which appear on axial slices as small ovoid or linear structures isointense to CSF, were quantified across four axial slices from the T2* GRE sequence. The four axial images used for analysis of ePVS corresponded to MRI slices containing the middle temporal lobe, basal ganglia and two slices from the centrum semiovale. Other sequences were consulted to rule out image artefacts or ePVS mimics. Two raters blinded to participant condition manually counted the number of ePVS in each slice. In the centrum semiovale, where most ePVS were observed in both mTBI patients and healthy controls, a categorical ePVS score of none (0 ePVS), mild (1–10 ePVS), moderate (11–20 ePVS), frequent (21–30 ePVS) or severe (>40 ePVS) was assigned to each slice as previously described.^17^

### Statistical analysis

Descriptive statistics were calculated for participant variables and are reported as mean with range. Normality was assessed with the Shapiro–Wilk test. ePVS are reported as mean and standard deviation, and between-group comparisons were performed with independent *t*-tests. Hedge’s *g* was used as a measure of effect size for between-group comparisons. Pearson’s correlations were conducted to analyse the hemispheric distribution of ePVS and relationship of ePVS with continuous participant variables. Simple linear regression was used to examine the relationship between ePVS number and number of lifetime TBIs in the patient cohort, with *η*^2^ reported as a measure of effect size. To determine inter-rater consistency in counting ePVS, the intra-class correlation coefficient was calculated based on a mean rating (*k* = 2), consistency and two-way mixed-effects model. All statistical analyses were performed with SPSS v27 (IBM, New York, USA), and the significance level was set at *α* = 0.05.

## Results

### Demographics, clinical, neurobehavioral and sleep measures

Nineteen participants (*n* = 11 mTBI; *n* = 8 healthy controls) were included in this analysis. With the exception of soft tissue swelling, all patients with mTBI had negative clinical CT scans. Structural MRI was negative for overt lesions in all but one patient, who had a small cluster of traumatic microbleeds in the frontal lobe. There was a significant age difference between mTBI patients and healthy controls (*P* = 0.05). mTBI participants scored significantly higher on the NSI relative to healthy controls across somatosensory, cognitive and affective domains (all *P* < 0.05). There was no significant difference in global PSQI scores between mTBI patients and healthy controls (*P* > 0.05). Participant characteristics, neurobehavioral symptoms and sleep data are presented in Table 1.

**Table 1:** Demographic, clinical, neurobehavioral and sleep characteristics of mTBI patients and healthy controls

	Healthy controls (*n* = 8)	mTBI (*n* = 11)	*P*-value
Age, years (range)	30.3 (21–44)	23.9 (18–36)	**0.05**
Previous # of TBIs (range)		2.45 (1–6)	
Hours to MRI (range)		64.6 (3.5–165)	
Mechanism of injury			
Motor vehicle Accident		3 (27.3%)	
Falls		4 (36.4%)	
Falls from height > 10 ft		1 (9.0%)	
Head struck object		3 (27.3%)	
Initial GCS (range)		13–15	
Sex			
Male	6 (75%)	6 (54.5%)	
Female	2 (25%)	5 (45.5%)	
Race			
White	6 (75%)	9 (81.8%)	
Asian	1 (12.5%)	2 (18.2%)	
NSI scores (SD)			
Somatosensory	1.3 (1.5)	8.8 (6.8)	**0**.**007**
Cognitive	1.1 (1.9)	5.9 (3.4)	**0**.**002**
Affective	2 (2.7)	6.8 (3.6)	**0**.**005**
Total	4.4 (3.7)	21.5 (12.1)	**0**.**001**
PSQI global score	4.6 (2.3)	6.1 (2.0)	0.148

Bold text indicates significant difference.

TBI, traumatic brain injury; GCS, Glasgow Coma Scale; NSI, Neurobehavioral Symptom Inventory; PSQI, Pittsburgh Sleep Quality Index.

### Enlarged perivascular spaces

**Figure 1:**
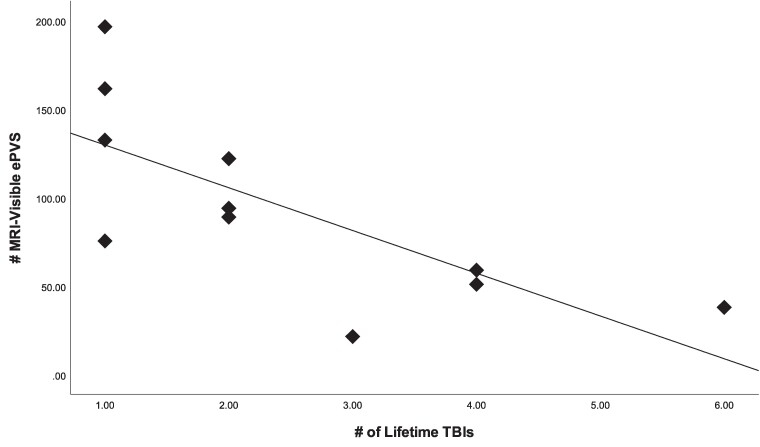
**Relationship between number of MRI-visible enlarged perivascular spaces (ePVS) and number of lifetime TBIs.** Each data point represents the number of MRI-visible ePVS in subjects with mTBI. ePVS number was inversely related to lifetime TBI exposure, assessed with linear regression. Those having more previous head injuries showed fewer MRI-visible ePVS (*β* = −0.73, *P* = 0.011).

**Figure 2:**
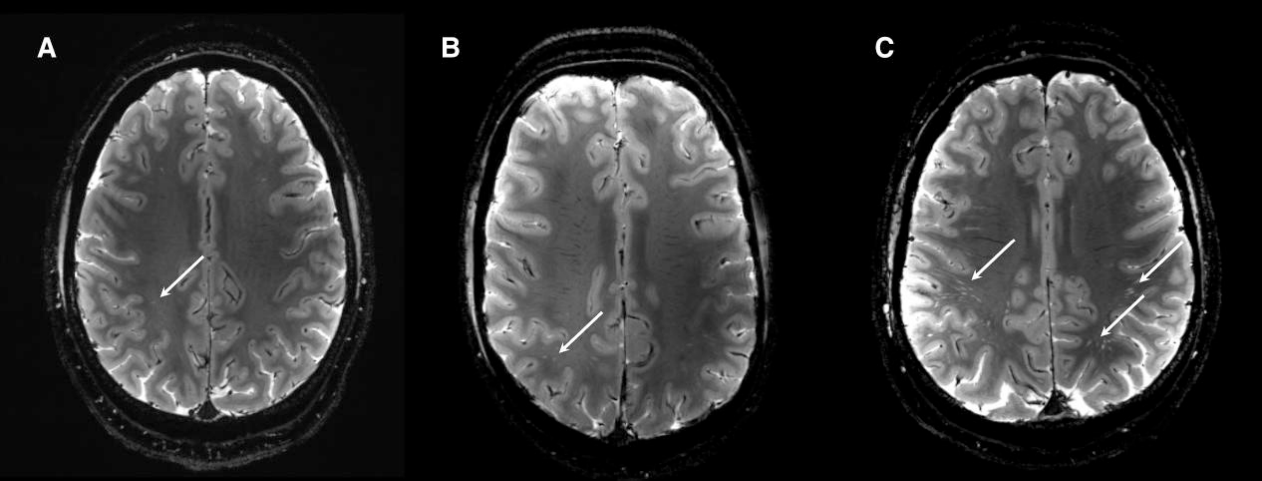
**Example MRIs of ePVS.** ePVS in the centrum semiovale in a healthy control participant (**A**), mTBI patient with a history of three previous head injuries (**B**) and mTBI patient with first-ever head injury (**C**). The healthy control participant and mTBI patient with previous head injuries received an ePVS grade of 1 (mild), while the patient with their first-ever mTBI received an ePVS grade of 4 (severe). Arrows indicate ePVS.

Normality of ePVS was assessed with Shapiro–Wilk and was found to be normally distributed, *W* (19) = 0.928, *P* = 0.16. There was excellent inter-rater consistency for the manual counts of ePVS (ICC = 0.96). ePVS were symmetrically distributed across hemispheres in both mTBI participants (*r* = 0.97, *P* = 0.015) and healthy controls (*r* = 0.90, *P* < 0.001). There was no significant relationship between age, sex and PSQI scores with ePVS in either healthy controls or mTBI patients. In healthy controls, there was a significant positive correlation between ePVS number and NSI total (*r* = 0.72, *P* = 0.04) and cognitive (*r* = 0.80, *P* = 0.02) scores. There were no significant correlations between ePVS number and NSI scores in mTBI patients. As shown in Table 2, the number of ePVS was significantly higher in mTBI patients than healthy controls (*P* = 0.015; *g =* 1.11). In both mTBI patients and healthy controls, the majority of ePVS were located in the centrum semiovale, with minimal ePVS located in other slices. Among healthy controls, six of the eight scored ‘none’ or ‘mild’ on the PVS rating scale, while two scored ‘moderate’. In mTBI patients, 10/11 TBI subjects scored ‘moderate’, ‘frequent’ or ‘severe’ (Table 2). The one mTBI patient who received a grade of ‘mild’ on the centrum semiovale slice had a history of five previous head injuries, while the two patients who scored ‘moderate’ had a history of two and three head injuries. Using linear regression, we found an inverse relationship between ePVS number and number of lifetime TBIs. As shown in Fig. 1, those patients with more lifetime TBIs exhibited fewer ePVS acutely after TBI (*β* = −0.71; *P* = 0.014; *η*^2^ = 0.53). Representative MR images of ePVS number in healthy controls and mTBI patients with and without a previous history of head injury are shown in Fig. 2.

**Table 2:** Quantitative and qualitative characteristics of ePVS

	Healthy controls (*n* = 8)	mTBI (*n* = 11)	*P*-value
# MRI-visible ePVS			
Mean (SD)	43.5 (32.2)	94.6 (53.9)	**0.015**
Range	9–94	21–197	
CSO ePVS rating			
None	2 (25%)		
Mild	4 (50%)	1 (9%)	
Moderate	2 (25%)	2 (18%)	
Frequent		6 (55%)	
Severe		2 (18%)	

Bold text indicates significant difference.

CSO, centrum semiovale; ePVS, enlarged perivascular space.

## Discussion

Consistent with previous research, our results suggest that mTBI results in acute alterations in perivascular space number. In our mTBI sample, ePVS were readily apparent on 7 T MRI during the acute stage of injury at a significantly higher rate than healthy controls. ePVS were distributed symmetrically across cerebral hemispheres, independent of location or mechanism of injury, and were not associated with measures of sleep. Lastly, we found that a history of repetitive head injury is associated with lower ePVS number at the acute post-injury stage.

The mechanisms underlying ePVS development under pathological conditions such as mTBI are currently unknown. Originally seen as incidental radiological findings, many studies have attempted to associate ePVS with neuropathology or symptom severity, with varying success. For instance, recent work has demonstrated that in patients with Alzheimer’s disease and cerebral amyloid angiopathy, a high burden of ePVS is associated with increased amyloid pathology.^7,18,19^ Thus, ePVS likely represent protein accumulation and stagnant fluid exchange under some pathological conditions. In moderate-severe TBI, proteins involved in neurodegeneration are known to accumulate rapidly,^20^ and there is evidence of microscopic multifocal axonal damage even in mTBI.^21^ Further, recent work in animal models has shown that TBI impairs clearance of interstitial solutes.^3,22^ Thus, ePVS observed during the acute stage of mTBI may represent rapid waste accumulation and impaired clearance along perivascular spaces. However, it remains unclear whether ePVS result from impaired waste clearance, or alternatively, waste clearance is impaired as a result of ePVS.^23^ Alternatively, it has been proposed that ePVS may reflect a compensatory response to facilitate waste clearance in response to injury or disease. Dai *et al*.^24^ used diffusion tensor imaging along the perivascular space (DTI-ALPS), a potential biomarker of glymphatic function, in 161 patients with mTBI. They observed increased glymphatic activity in patients with mTBI relative to healthy controls. This effect was most pronounced in younger subjects, and these authors proposed this could reflect a compensatory response to facilitate waste clearance.

Not all studies have found a relationship between ePVS and TBI. For example, Opel *et al*.^13^ did not find a significant difference in ePVS number, and Hicks *et al*.^12^ found no difference in either PVS number or volume, between TBI patients and controls. Two studies which utilized both manual counts of ePVS as well as DTI-ALPS found significant differences with the ALPS index, but not manual counts, between healthy controls and TBI patients.^25,26^ While this could reflect the sensitivity of each methodology, it is also worth noting that the methods used to quantify PVS are substantially different from one study to another. While ePVS visible on T2-weighted MRI are likely periarterial in nature, DTI-ALPS measures perivenous diffusivity in a relatively small periventricular area. A recent critique has highlighted that segmentation techniques used to quantify PVS are highly variable and inconsistent, even when the same segmentation techniques are used.^27^ Thus, we chose a visual rating scale which has been validated across a number of studies and clinical populations for quantifying ePVS. MRI field strength will also affect the number of observed PVS, which likely explains why our study using 7 T MRI found a higher number of ePVS compared to other studies, even those that quantified whole-brain PVS. It is currently unclear the extent to which ePVS observed after TBI are permanent changes. The initial report of PVS in mTBI suggested that these changes may be permanent,^11^ yet no studies to date have performed serial imaging of PVS in the same cohort.

Few studies have examined the relationship between ePVS number and repeat mTBI. In a previous study of veterans with mTBI examined 55 months post-injury, both ePVS number and volume were positively correlated with the number of mTBIs experienced in military service.^14^ However, we found that the previous number of TBIs was negatively correlated with ePVS number during the acute stage of injury. The contrasting results from our study may be due to several factors. First, Piantino *et al*.^14^ studied veterans with a reported history of mTBI, including possible blast injury. Previous studies indicate that the mechanism of injury in military TBI may produce distinct pathophysiology from that of blunt head trauma.^28^ Importantly, the timing of MRI post-injury likely plays an important role in characterizing ePVS in patients with mTBI. Most studies examining ePVS in patients with mTBI have been conducted months or years following injury. ePVS number in the acute versus chronic stages of injury may be due to different underlying mechanisms. Thus, one possible explanation for the data observed in this study is that mTBI elicits a compensatory increase in ePVS as a response to injury, and this response becomes diminished after multiple TBIs. Interestingly, a similar pattern of results has been observed in astronauts following long-duration spaceflight. Novice astronauts showed an increase in ePVS volume during a 6-month mission to space, whereas experienced astronauts who had completed multiple space missions did not exhibit this response.^29^

Disrupted sleep is another mechanism by which ePVS may increase following mTBI. Piantino *et al*.^14^ found that patients identified as poor sleepers showed a greater increase in ePVS volume for each subsequent mTBI, compared to those identified as good sleepers. Other studies have demonstrated a relationship between ePVS, total sleep time and sleep efficiency in patients with TBI.^13^ These findings should not be surprising, as impaired sleep has been associated with a high ePVS burden even in non-clinical samples,^30^ and glymphatic function is thought to be closely tied to the sleep cycle.^31^ Furthermore, impaired sleep is among the most commonly reported symptoms following TBI.^32^ In our study, there were no significant differences in PSQI scores between mTBI patients and healthy controls and no significant relationship between ePVS and total sleep time or sleep efficiency in either patients or controls. Thus, while impaired sleep may contribute to ePVS in subacute to chronic TBI, it is not a likely explanation for the greater ePVS number seen in mTBI patients in this acute study. Finally, although the age of healthy controls was significantly higher than that of mTBI patients, this should not alter the pattern of results seen in this study, as ePVS number generally increases with age.^33^

This study has several limitations. First, the sample size is small, and additional prospective studies with larger sample sizes are needed to replicate these findings. Second, we relied on manual counts to quantify ePVS. Although more sophisticated techniques to quantify ePVS have been proposed,^27^ our measures of internal consistency were high, and the qualitative rating scale used here has been validated in numerous studies. Third, the mean age of our sample is quite young. These results may not be representative of the general population but may rather reflect ePVS characteristics following mTBI in young adults. Fourth, we relied on the OSU-TBI form to quantify previous head injury history. While the medical record may be better for identifying head injuries resulting in hospitalization, we note that many head injuries do not result in individuals seeking medical care and are thus not contained in the medical record. Furthermore, the reliability and predictive validity of the OSU-TBI form have been established previously.^34,35^ Lastly, there is some controversy about using ePVS to make inferences about perivascular waste clearance and glymphatic function. Specifically, it is unclear whether deep white matter ePVS are truly representative of the brains overall glymphatic activity.^36^ Caution is thus warranted when making inferences about glymphatic function based on ePVS.

## Conclusions

Taken together with previously published work, the results of this study suggest that ePVS are common imaging markers in mTBI that appear rapidly after injury. The diminished ePVS number in patients with a history of multiple mTBIs is an important finding and further underscores that repetitive head injury may be associated with distinct neuropathology. The current assumption in most studies is that ePVS are a surrogate marker of glymphatic function, although this has not been shown definitively in mTBI. Our findings raise the possibility that patients with multiple head injuries, who are most likely to develop pathologies such as chronic traumatic encephalopathy,^37^ have a unique ePVS response to injury. Currently, it is unclear how the ePVS number changes over time in individual patients, as no studies to date have examined ePVS longitudinally. In addition to neuropathology, post-traumatic symptoms including impaired sleep, headache and mood disorder may contribute indirectly to ePVS in the chronic stage of injury. The relationship between ePVS and neuropsychological or neurobehavioral outcome is also unclear. A few studies have shown a relationship between ePVS and various neuropsychological measures in chronic mTBI, but future prospective studies with comprehensive clinical and neuropsychological outcomes are warranted. Although there is no single treatment for symptoms of mTBI, imaging biomarkers such as ePVS may help identify those patients who can benefit from early and targeted intervention.

## Data Availability

De-identified data not published in this article will be made available upon reasonable request from any qualified investigator.
